# Building an Equity‐Centered Nursing Leadership Workforce: A Framework for Transformative Change

**DOI:** 10.1111/jan.17097

**Published:** 2025-06-03

**Authors:** Suha Ballout

**Affiliations:** ^1^ Manning College of Nursing and Health Sciences, University of Massachusetts Boston Boston Massachusetts USA

**Keywords:** critical race theory, health equity, nursing leadership, policy advocacy, transformative leadership, workforce diversity

## Abstract

**Background:**

The nursing workforce is central to healthcare delivery, yet racial and ethnic disparities persist. Systemic barriers such as implicit bias, institutional racism, and socioeconomic obstacles hinder the career progression of underrepresented nurses. Addressing these disparities is critical to fostering a diverse workforce that can effectively meet the needs of diverse patient populations.

**Aim:**

This paper introduces the Equity‐Centered Nursing Leadership Framework (ECNLF), a structured approach to advancing diversity in the nursing workforce. The framework provides strategies for dismantling systemic inequities and establishing sustainable leadership pathways.

**Design:**

Conceptual and theoretical analysis of nursing leadership and workforce equity.

**Methods:**

A targeted literature synthesis of peer‐reviewed research, policy reports, and theoretical frameworks was conducted. The ECNLF was conceptualised using an integrative approach that examines systemic inequities in nursing leadership and workforce diversity. This framework identifies structural barriers, proposes equity‐driven interventions, and outlines strategies for mentorship, leadership development, and policy advocacy.

**Results:**

The ECNLF provides a structured approach for integrating equity‐driven leadership development into nursing education and practice. Its implementation is guided by three key pillars: structured mentorship, leadership development, and policy advocacy. The framework supports the establishment of formal mentorship programmes, ensuring that underrepresented nurses have access to career advancement opportunities and leadership training.

**Conclusion:**

Systemic change requires integrating equity frameworks into nursing curricula, adopting structured DEI initiatives in healthcare institutions, and expanding policy‐driven funding for leadership development. The ECNLF provides a scalable model for reducing workforce disparities, improving retention, and fostering inclusive nursing leadership that reflects and serves diverse communities.

## 1 Introduction

The nursing workforce plays a critical role in healthcare delivery, serving as the backbone of patient care across various settings. Despite its significance, disparities in workforce diversity persist, limiting representation among racial and ethnic minority groups (Hynson et al. 2022; Kozhimannil et al. 2021). Currently, approximately 80% of registered nurses in the United States identify as White/Caucasian, even though this group comprises only 61.3% of the general population (American Association of Colleges of Nursing 2022). African American nurses account for only 6.3% of the workforce despite a greater population representation (Iheduru‐Anderson 2020; Hynson et al. 2022). In countries such as Canada, the UK, and Australia, similar trends in the underrepresentation of racial and ethnic minorities in the nursing workforce exist (Harrison and Hauck 2023; Phillips et al. 2021). A diverse nursing workforce is essential in addressing health inequities, improving cultural competence, and enhancing patient‐centred care (Takeshita et al. 2020). This paper examines the Equity‐Centered Nursing Leadership Framework (ECNLF), a model developed within the Clinical Leadership Collaborative for Diversity in Nursing (CLCDN), a partnership between a public university's college of nursing and a major healthcare employer aimed at addressing workforce disparities through mentorship, policy advocacy, and leadership training.

## 2 Background

### 2.1 Workforce Diversity Gaps in Nursing

Although recent workforce data indicate gradual progress, racial and ethnic disparities remain significant. According to the 2022–2023 National Sample Survey of Registered Nurses (NSSRN), Black nurses represent 11% and Asian nurses 9% of the RN workforce; an increase from 2018, but still disproportionately low compared to national demographics (Health Resources and Services Administration [HRSA] 2024a, 2024b). This underrepresentation highlights persistent systemic barriers in recruitment, retention, and leadership advancement (McHugh and Ma 2014). Furthermore, workplace conditions and job satisfaction among nurses have declined, emphasising the need for systemic changes to improve retention, equity, and leadership development for minority nurses (HRSA 2024a, 2024b). This workforce challenge is compounded by the increasing demand for nurses. The Bureau of Labor Statistics (BLS) projects that the RN workforce will grow from 3.1 million in 2022 to 3.3 million in 2032, with about 193,100 RN job openings annually due to retirements and workforce exits (BLS 2024). This demand further underscores the urgency of enhancing recruitment, retention, and leadership opportunities for underrepresented nurses to ensure a sustainable, diverse workforce (American Association of Colleges of Nursing [AACN], n.d.). This underrepresentation in nursing roles perpetuates health disparities and limits opportunities for minority nurses (Kozhimannil et al. 2021). Systemic challenges, including implicit bias, institutional racism, and socioeconomic barriers, continue to hinder workforce diversity (Iheduru‐Anderson and Alexander 2022).

### 2.2 Barriers to Workforce Diversity

The workforce disparities are the result of historical trends that perpetuate deeply ingrained systemic challenges. Structural barriers, including implicit bias in hiring, exclusion from professional networks, and financial obstacles, limit career progression for underrepresented nurses (Iheduru‐Anderson and Alexander 2022; Kozhimannil et al. 2021). Policy‐driven solutions such as bias training, equity‐focused recruitment, and tuition support programmes have shown effectiveness in reducing these disparities (Marcelin et al. 2019; Urban Institute 2022). Programmes such as the CLCDN demonstrated the effectiveness of structured mentorship, leadership training, and policy advocacy in increasing diversity in nursing leadership roles (Banister et al. 2020). By integrating these policies and interventions, nursing can move toward an equitable, diverse, and inclusive workforce that reflects the communities it serves.

Research consistently demonstrates the substantial benefits of a diverse nursing workforce, extending beyond mere demographic representation (Carter 2020; Gomez and Bernet 2019). Studies have shown that healthcare organisations with diverse nursing teams exhibit higher levels of cultural competence, improved patient satisfaction, and better health outcomes across diverse patient populations (Takeshita et al. 2020). Also, a diverse nursing workforce is better equipped to address health disparities and effectively respond to the unique needs of underserved communities, ultimately leading to enhanced healthcare quality and improved patient outcomes (Guglielminotti et al. 2022). University‐healthcare partnerships designed to promote workforce diversity have been shown to improve minority representation in healthcare leadership and enhance patient‐centred care (Iheduru‐Anderson and Alexander 2022). Hence, there is an urgent need to establish innovative programmes that increase workforce diversity and equip nurses with the skills needed to manage the modern‐day complex healthcare issues for a majority diverse patient population (Noone et al. 2020). As James Baldwin (1962) wrote, “Not everything that is faced can be changed, but nothing can be changed until it is faced.” As such, there is a need to confront systemic inequities in nursing leadership and education to foster meaningful change.

While these disparities persist, there is growing recognition that structural interventions are necessary to create meaningful change. Amidst these challenges, initiatives like the CLCDN emerge as beacons of progress and innovation (Banister et al. 2014; Banister et al. 2020). Spearheaded by a partnership between a major public university college of nursing and the largest healthcare employer in the state, the CLCDN represents a proactive effort to address disparities in nursing workforce diversity (Guglielminotti et al. 2022). Through strategic collaborations, partnerships, and targeted interventions, the CLCDN aims to diversify the nursing workforce and nurture the leadership potential of underrepresented groups. At the heart of the CLCDN's mission is integrating academic rigour with real‐world practice, fostering symbiotic relationships between educational institutions and healthcare organisations (Carter 2020). The CLCDN prepares diverse nursing students to excel in clinical settings and contribute to healthcare excellence by providing opportunities for leadership development, mentorship, and scholarly engagement. Also, by establishing a network of support and mentorship, the CLCDN lays the foundation for sustainable change, ensuring that future generations of nurses have the resources and guidance they need to succeed. Ultimately, “the classroom remains the most radical space of possibility in the academy” (Hooks 1994).

For example, the success story of SD, a graduate of the CLCDN programme, illustrates the transformative impact of equity‐centered leadership training. SD's journey from a nursing student to a leadership role within a major healthcare institution demonstrates how the programme equips underrepresented nurses with the skills and confidence to excel in their careers. This experience aligns with research emphasising the role of structured mentorship and leadership programmes in increasing retention and professional advancement among minority nurses (Iheduru‐Anderson and Alexander 2022). So, initiatives like the CLCDN exemplify the transformative potential of equity‐centered nursing leadership. By prioritising diversity, equity, and inclusion within the nursing profession, we uphold the principles of social justice and pave the way for a future where healthcare is accessible and equitable for all. Through collaborative efforts and unwavering commitment, we can bridge the gaps in nursing workforce diversity, thereby realising the full potential of a healthcare system that serves and uplifts every member of our diverse society.

This article aims to discuss the conceptualization of the Equity‐Centered Nursing Leadership Framework that guided the success of the CLCDN programme. Sharing this framework is critical for fostering sustainable diversity and equity initiatives in nursing. By illustrating practical strategies that enhance mentorship, leadership development, and institutional support, this framework serves as a replicable model for nursing schools and healthcare organisations. Also, it highlights the role of policy advocacy, systemic change, and educational reform in creating sustainable pathways for underrepresented nurses to advance in their careers. By implementing similar models, stakeholders can cultivate a more inclusive workforce, ultimately improving patient care outcomes and advancing health equity on a broader scale.

## 3 Data Sources

This discussion paper is grounded in theoretical analysis and conceptual development, informed by critical frameworks that address nursing workforce equity, leadership, and systemic barriers. The Equity‐Centered Nursing Leadership Framework is constructed through a synthesis of Black feminist thought, critical race theory, intersectionality, social determinants of health, and transformative leadership theory. A targeted literature review was conducted using PubMed, CINAHL, Scopus, and Google Scholar, focusing on peer‐reviewed publications, policy reports, and theoretical papers from 2000 to 2024. Search terms included “nursing leadership diversity,” “equity in nursing education,” “critical race theory in healthcare,” and “mentorship and retention of minority nurses.” This discussion is not based on primary empirical data but synthesises theoretical perspectives and literature‐based insights to advance a new conceptual model for nursing leadership and workforce equity.

## 4 Discussion

### 4.1 Theoretical Foundations of the Equity‐Centered Nursing Leadership Framework

**FIGURE 1:**
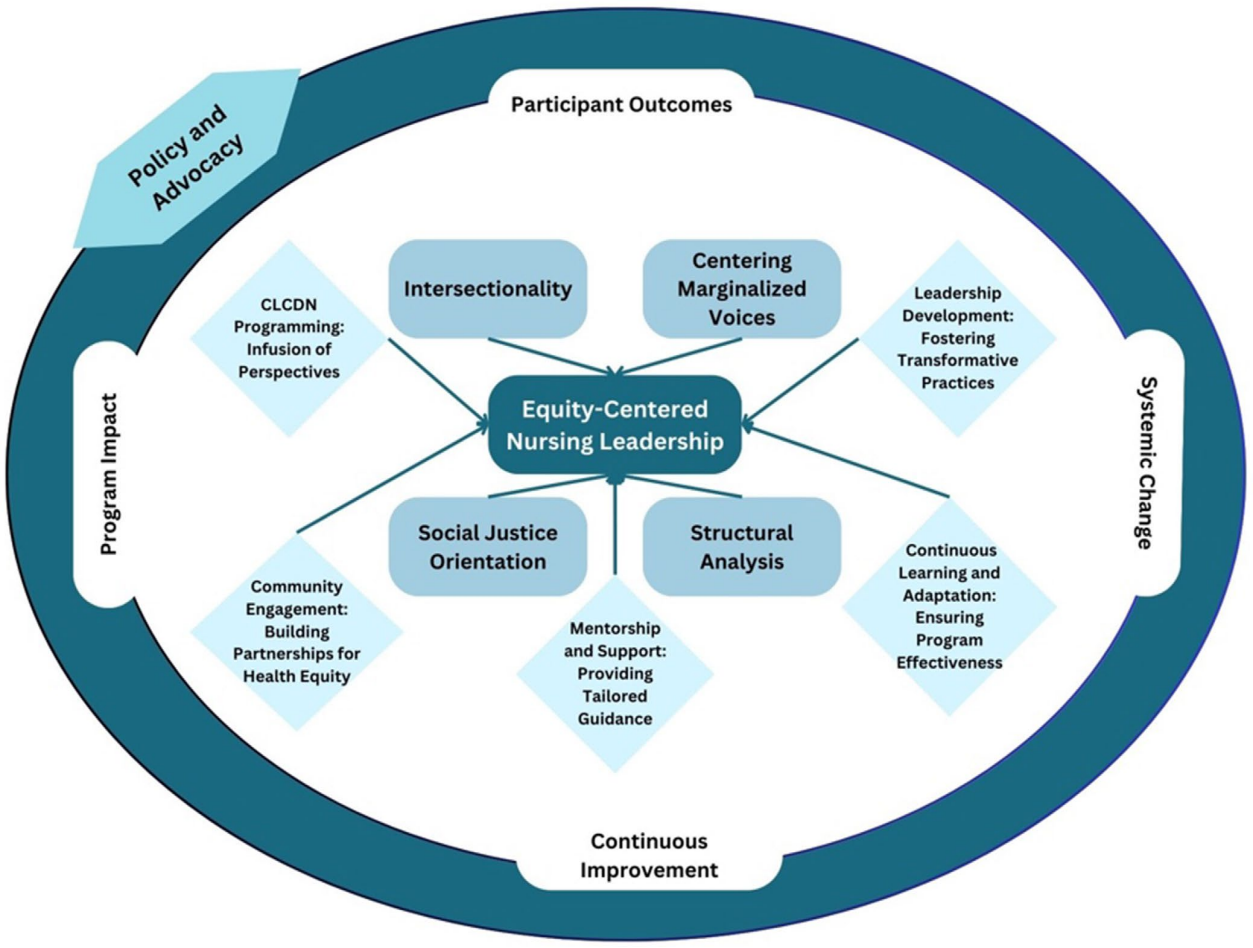
Equity centered nursing leadership conceptual framework.

The Equity‐Centered Nursing Leadership Framework (ECNLF) (Figure 1) integrates multiple theoretical perspectives to address systemic inequities in nursing education and practice. These theoretical underpinnings, Black feminist thought (Hill Collins 2000; De Sousa and Varcoe 2022), critical race theory (Ladson‐Billings and Tate 1995; Iheduru‐Anderson and Alexander 2022), intersectionality (Crenshaw 1989; Aspinall et al. 2023), social determinants of health framework (Davis and Lindell 2024; Alasiri et al. 2024), and transformative leadership theory (Shields 2010; Nikpour et al. 2022), provide an integrated lens for understanding barriers to leadership and guiding systemic change in the nursing profession. The ECNLF is rooted in critical theories of oppression, drawing from Black feminist thought, critical race theory, and intersectionality to examine how racism, sexism, and systemic exclusion shape the experiences of underrepresented nurses (Hill Collins 2000; Crenshaw 1989; De Sousa and Varcoe 2022). Black feminist thought highlights the marginalisation of Black women and other minority groups, centring their lived experiences in leadership development (Hill Collins 2000). Critical race theory builds on this foundation, showing how systemic racism is embedded in nursing education, hiring, and promotion, necessitating anti‐racist policies and structural reform (Ladson‐Billings and Tate 1995; Iheduru‐Anderson and Alexander 2022). Intersectionality further explains how race, gender, and socioeconomic factors intersect, requiring leadership pathways that account for the unique barriers faced by nurses from diverse backgrounds (Crenshaw 1989; Aspinall et al. 2023). The social determinants of health framework complements this analysis by linking structural barriers such as unequal access to education, economic disparities, and professional exclusion to workforce diversity gaps (Alasiri et al. 2024). Transformative leadership theory provides a model for reshaping institutional norms, advocating for inclusive mentorship, policy‐driven change, and systemic accountability (Nikpour et al. 2022).

Integrating critical theories of oppression with leadership and workforce development models, the ECNLF provides a structured approach to dismantling systemic barriers and fostering an equitable, diverse, and inclusive nursing profession (Hill Collins 2000; Crenshaw 1989; Shields 2010). This multi‐theoretical framework informs policy change and institutional accountability and creates tangible leadership pathways for historically excluded nurses, ensuring that workforce equity remains a sustained and measurable priority in nursing education and healthcare leadership (Iheduru‐Anderson and Alexander 2022; Nikpour et al. 2022). Unlike traditional nursing leadership models, which often emphasise individual skill‐building and clinical expertise, the ECNLF centres on structural transformation (Shields 2010). While many existing leadership programmes focus on professional development, ECNLF actively addresses systemic inequities in hiring, promotions, and mentorship access (De Sousa and Varcoe 2022; Bailey et al. 2017). By integrating policy reform, anti‐bias training, and leadership pipelines, ECNLF ensures that diversity efforts are institutionalised rather than performative (Phillips et al. 2021; The Joint Commission (TJC) 2023).

### 4.2 Practical Implementation: Barriers and Challenges in Nursing Leadership

#### 4.2.1 Current Challenges in Nursing Diversity

While these theoretical perspectives offer a strong foundation for the Equity‐Centered Nursing Leadership Framework, real‐world barriers must be addressed to ensure effective implementation. Unlike conventional nursing leadership models, the ECNLF does not merely prepare individual nurses for leadership; it creates structural mechanisms that sustain equity‐centered mentorship, career advancement, and anti‐bias policies within institutions (Phillips et al. 2021). Despite incremental progress, workforce disparities persist due to deeply ingrained structural barriers (Hynson et al. 2022). Research highlights implicit bias in hiring and promotions, workplace exclusion from leadership networks, and limited access to mentorship opportunities, all of which disproportionately affect minority nurses (Iheduru‐Anderson and Alexander 2022; De Sousa and Varcoe 2022). Institutional racism is reflected in restrictive workplace cultures, where underrepresented nurses face barriers to leadership training and career advancement (Bailey et al. 2017). At the systemic level, the financial burden of higher education, lack of executive training access, and socioeconomic disadvantages further restrict leadership pathways (Alasiri et al. 2024). Overcoming these challenges requires a dual approach: institutional change through mentorship programmes, anti‐bias training, and career development initiatives, alongside policy interventions to expand scholarship funding, leadership training fellowships, and equity‐driven accreditation standards (HRSA, 2024; TJC 2023).

#### 4.2.2 Implicit Bias, Institutional Racism, and Socioeconomic Barriers

Systemic barriers continue to hinder the progress of representation within the nursing workforce, reinforcing inequities in hiring, promotion, and professional development opportunities (Phillips and Malone 2014; Noone and Villarruel 2021). These barriers operate at two distinct levels: institutional and systemic. Institutional barriers emerge within healthcare organisations, directly affecting workplace culture and career advancement (Hall et al. 2015). Systemic barriers, on the other hand, reflect broader structural inequities in education, economic opportunity, and access to leadership pathways (Narayan 2019; Noone et al. 2020). Addressing these challenges requires targeted interventions that integrate diversity‐focused recruitment, leadership mentorship, and policy‐driven accountability measures to ensure equitable career advancement opportunities for underrepresented nurses (Bailey et al. 2017).

Implicit bias remains pervasive in nursing institutions, shaping hiring decisions, performance evaluations, and leadership selection processes (De Sousa and Varcoe 2022). Research highlights how unconscious biases influence perceptions of leadership potential, often resulting in minority nurses being overlooked for promotions despite their qualifications (Iheduru‐Anderson and Alexander 2022). These biases limit career progression and create workplace climates where underrepresented nurses face greater scrutiny, less recognition, and fewer opportunities for leadership development (De Sousa and Varcoe 2022). Also, institutional racism is embedded in workplace policies and professional networks, restricting access to mentorship, sponsorship, and career‐building opportunities (Iheduru‐Anderson 2020). Many underrepresented nurses experience exclusion from informal networks of influence, where leadership opportunities are often discussed and facilitated (De Sousa and Varcoe 2022). This lack of access to professional support systems perpetuates disparities, making it difficult for minority nurses to advance within healthcare institutions.

Beyond institutional challenges, systemic barriers rooted in economic and educational disparities create obstacles that prevent underrepresented nurses from entering and excelling in leadership roles (Alasiri et al. 2024). The financial burden of higher education and leadership training disproportionately affects nurses from historically marginalised backgrounds, limiting access to graduate education, executive training programmes, and professional development resources (Davis and Lindell 2024). Many aspiring minority nurse leaders face systemic disadvantages such as lower‐income backgrounds, restricted access to scholarships, and limited institutional financial support, making career advancement more difficult (Alasiri et al. 2024). Also, mentorship gaps further compound these systemic inequities. Minority nurses frequently lack access to sponsorship and advocacy networks that help navigate complex career pathways, obtain leadership training, and secure executive roles (Phillips et al. 2021). Without targeted interventions such as structured mentorship programmes, tuition assistance, and policy‐driven hiring reform, these systemic barriers will continue to restrict leadership opportunities for diverse nurses.

Addressing these challenges requires a dual approach that targets both institutional and systemic barriers. Institutionally, nursing organisations need to implement anti‐bias training, mentorship structures, and equitable promotion pathways to create an environment where all nurses, regardless of background, have the opportunity to thrive in leadership roles (Hall et al. 2015; Banister et al. 2020). Systemically, expanding financial aid programmes, leadership fellowships, and targeted recruitment efforts can help eliminate structural inequities in access to higher education and professional development (HRSA, 2024; Noone et al. 2020). By aligning institutional reform with systemic policy interventions, nursing institutions and policymakers can create sustainable pathways for underrepresented nurses to ascend into leadership roles, ensuring a diverse and inclusive nursing workforce (Iheduru‐Anderson and Alexander 2022; TJC 2023).

### 4.3 Policy Interventions for Advancing Workforce Diversity

Systemic inequities in nursing leadership cannot be addressed without targeted policy interventions that support workforce diversity. In the United States, federal and institutional policies play a pivotal role in addressing workforce diversity gaps. The Title VIII Nursing Workforce Diversity Programme funds recruitment, training, and leadership development for underrepresented nurses, resulting in higher retention rates and career advancement outcomes (HRSA, 2024). Similarly, the Joint Commission (TJC) and the Magnet Recognition Programme enforce diversity standards, requiring implicit bias education, cultural competency training, and equity‐focused hiring practices as part of hospital accreditation (TJC 2023; ANCC 2023). At the institutional level, successful models such as Mass General Brigham's Disparities Solutions Centre demonstrate how integrating bias training, leadership coaching, and mentorship pathways leads to higher representation of minority nurses in leadership roles (Bailey et al. 2017). Scaling such evidence‐based strategies across healthcare institutions is essential for long‐term workforce transformation.

The Equity‐Centered Nursing Leadership Framework (ECNLF) aligns with and can further enhance global efforts to promote workforce diversity in nursing leadership. Countries such as the United Kingdom, Canada, and Australia have implemented policies aimed at increasing representation and reducing disparities in nursing leadership. For example, the UK's National Health Service (NHS) Workforce Race Equality Standard (WRES) mandates data reporting on leadership diversity and enforces accountability measures to address inequities (NHS England 2023). Similarly, Canada's Nursing Leadership Framework emphasises equity‐driven mentorship programmes to support Indigenous and minority nurses in leadership roles (Canadian Nurses Association 2023). In Australia, the Emerging Nurse Leader Programme, led by the Australian College of Nursing, provides structured support for underrepresented nurses pursuing leadership pathways (Australian College of Nursing 2023). By integrating the ECNLF within these international initiatives, healthcare systems can adopt a more comprehensive, evidence‐based approach to dismantling structural barriers, expanding mentorship networks, and institutionalising equity in nursing leadership worldwide. Adapting ECNLF principles to global nursing contexts can help create sustainable, systemic change that fosters a more representative and inclusive workforce.

### 4.4 Moving Forward: The Role of the Equity‐Centered Nursing Leadership Framework

Addressing systemic barriers in nursing requires a structured, theory‐driven approach that translates conceptual insights into practical, sustainable interventions. The ECNLF operationalises equity‐driven leadership by embedding mentorship, training, and systemic policy reform into nursing education and workforce development. Unlike traditional leadership models, the ECNLF actively counters structural inequities through anti‐bias hiring initiatives, diversity leadership pipelines, and community‐driven partnerships (Nikpour et al. 2022). Pilot programmes such as CLCDN have demonstrated measurable increases in minority nurse leadership representation, validating the effectiveness of structured mentorship and career development initiatives (Banister et al. 2020). Scaling this model across healthcare institutions requires collaboration between nursing schools, policymakers, and hospital administrators. The ECNLF serves as a replicable framework, offering a roadmap for sustained workforce equity, increased patient‐centred care, and improved leadership outcomes in nursing.

By bridging theoretical foundations with practical implementation, the ECNLF ensures that diversity, equity, and inclusion efforts extend beyond conceptual discourse and result in systemic, measurable change. Through initiatives like the CLCDN and other equity‐centered workforce development programmes, the framework offers a replicable model for nursing schools, healthcare institutions, and policymakers seeking to build an inclusive workforce that reflects the diverse populations it serves. Ensuring the widespread adoption of this framework will be critical in advancing health equity, improving patient outcomes, and fostering a more representative and empowered nursing leadership.

### 4.5 Equity‐Centered Nursing Leadership Framework: Programme Implementation

While policy interventions establish the foundation for workforce diversity, effective implementation requires a structured framework. The ECNLF operationalises these principles through targeted leadership training, mentorship, and community partnerships. Research indicates that structured frameworks can drive meaningful change toward inclusivity in nursing leadership (Dreachslin et al. 2017). For instance, implementing organisational frameworks that prioritise diversity, equity, inclusion, and belonging has been shown to foster cultural competency and inclusive environments within nursing teams (American Organization for Nursing Leadership 2024). Mentorship programmes are a critical component of such frameworks, enhancing leadership awareness, motivation, stress‐coping mechanisms, and confidence among participants (Bynum et al. 2023). The implementation of the ECNLF is structured around five key areas: leadership development, community engagement, culturally and linguistically congruent diversity nursing (i.e., CLCDN) programming, mentorship and support, and continuous learning and adaptation. By focusing on these areas, the ECNLF aims to create an environment where all nurses, regardless of background, have the opportunity to thrive in nursing roles, thereby promoting a diverse and inclusive workforce.

Leadership development is a cornerstone of the ECNLF, directly addressing systemic barriers that limit underrepresented nurses' access to leadership roles. The programme applies transformative leadership principles (Nikpour et al. 2022) to equip nurses with the skills needed to navigate structural inequities, advocate for systemic reforms, and lead within diverse healthcare settings. The leadership training model integrates formal leadership professional development, mentorship, and other opportunities that empower nurses from underrepresented backgrounds to advance their careers and assume nursing positions. Programmes such as coaching, professional development workshops, and institutional mentorship initiatives provide structured pathways for emerging nurse leaders to develop competencies in decision‐making, strategic planning, and policy advocacy (Iheduru‐Anderson and Alexander 2022). These efforts are crucial, as implicit bias and institutional hiring practices often create barriers to leadership advancement for minority nurses (De Sousa and Varcoe 2022). Also, structured networking opportunities play a critical role in advancing leadership equity. The CLCDN programme facilitates mentorship partnerships between emerging nurse leaders and experienced professionals who provide career guidance, sponsorship, and professional advocacy (Banister et al. 2020). These initiatives foster transformative leadership by creating support systems that counteract the systemic challenges faced by underrepresented nurses in leadership progression.

A core strategy of the ECNLF is its emphasis on community partnerships as a mechanism for fostering equitable healthcare systems and leadership development. Engaging with community organisations, healthcare institutions, and academic partners allows the ECNLF to expand its impact beyond individual leadership development and integrate structural interventions aimed at addressing social determinants of health (Alasiri et al. 2024). Community engagement efforts include collaborative initiatives between nursing schools, hospitals, and other agencies, ensuring that leadership training aligns with the needs of underserved populations. Programmes that include community‐based service‐learning experiences and policy‐driven advocacy training provide nursing students and professionals with hands‐on experiences in addressing healthcare disparities while simultaneously enhancing their leadership skills. The CLCDN programme exemplifies this collaborative approach by partnering with a major local health system, professional nursing organisations, and advocacy groups to provide leadership training that incorporates cultural competence, anti‐racist frameworks, and community‐centred care (Bailey et al. 2017). By embedding community engagement into leadership development, the ECNLF ensures that emerging nurse leaders are equipped to enact systemic change both within healthcare institutions and the broader public health sector.

The Clinical Leadership Collaborative for Diversity in Nursing (CLCDN) serves as a model for operationalising the ECNLF by integrating transformative leadership training, mentorship, and systemic policy advocacy. The programme is structured to bridge the gap between academic preparation and workforce leadership, ensuring that underrepresented nursing students and professionals receive targeted support in career progression and leadership development. By embedding Black feminist thought, critical race perspectives, and intersectionality frameworks into its programming, the CLCDN prepares nursing students and emerging leaders to critically assess and challenge systemic inequities (De Sousa and Varcoe 2022; Crenshaw 1989). Workshops, guest lectures, and mentorship forums provide students with direct engagement with diverse nursing leaders who model equity‐centered leadership practices (Nikpour et al. 2022). One of the key successes of the CLCDN is its measurable impact on workforce diversity. Data indicate that graduates of the programme demonstrate higher retention rates, greater leadership representation, and stronger advocacy skills in promoting workforce equity (Iheduru‐Anderson and Alexander 2022). The programme's emphasis on financial support, policy education, and leadership pathways has been linked to improved career mobility among underrepresented nurses, reinforcing its role as a best‐practice model for workforce diversity initiatives (Guglielminotti et al. 2022).

Mentorship is a foundational element of the ECNLF, ensuring that underrepresented nurses receive personalised guidance, professional sponsorship, and career advocacy. The mentorship model is designed to address both the structural barriers and the individualised career challenges faced by minority nurses (Iheduru‐Anderson and Alexander 2022). The CLCDN and other ECNLF‐aligned programs emphasise structured mentorship initiatives, pairing early‐career nurses with experienced leaders who provide career coaching, strategic networking, and professional sponsorship. This structured approach ensures that mentees receive targeted support in overcoming workplace biases, navigating leadership pathways, and advocating for institutional change (Banister et al. 2020). Beyond traditional mentorship, the ECNLF also integrates peer‐to‐peer support networks that foster community, collaboration, and knowledge‐sharing among underrepresented nurses. Professional development workshops focus on career advancement strategies, self‐care, and leadership resilience, equipping nurses with the tools to navigate systemic challenges and sustain long‐term leadership success.

A critical component of the ECNLF is its commitment to continuous assessment and refinement to ensure long‐term effectiveness and sustainability. Given that institutional resistance and systemic inequities evolve over time, the framework incorporates regular evaluation mechanisms to measure its impact and adapt programming to emerging challenges (Nikpour et al. 2022). Key evaluation strategies include longitudinal data tracking on leadership retention, career progression, and workforce equity metrics. Institutions implementing ECNLF‐driven programmes conduct equity audits, participant feedback assessments, and diversity impact reports to ensure that the programme's initiatives translate into measurable workforce transformation (Alasiri et al. 2024). Ongoing professional development opportunities such as bias training, leadership coaching, and policy engagement workshops ensure that ECNLF programmes remain responsive to the needs of both nursing students and professionals (Hall et al. 2015).

The ECNLF provides a structured, evidence‐based approach to addressing longstanding disparities in nursing leadership and workforce diversity. Table 1 provides a stepwise process that institutions can adopt to integrate equity‐centered leadership initiatives within nursing education and workforce development. By integrating transformative leadership development, community engagement, structured mentorship, and continuous evaluation, the framework creates a replicable model for advancing health equity in nursing. The success of pilot programmes like the CLCDN demonstrates the tangible impact of equity‐centered leadership training in increasing the representation of underrepresented nurses in leadership roles. Moving forward, the scalability of ECNLF‐aligned initiatives across healthcare institutions and academic settings will be critical to ensuring long‐term systemic change. By sustaining mentorship networks, leadership pathways, and policy‐driven interventions, the ECNLF holds the potential to reshape the nursing profession into one that truly reflects the diverse communities it serves.

**TABLE 1:** Implementation steps for the Equity‐Centered Nursing Leadership Framework (ECNLF).

ECNLF implementation step	Key actions	Expected outcomes
Step 1: Institutional Commitment	Establish leadership buy‐in, integrate DEI goals into strategic plans, and align with accreditation standards.	Organisational culture shift toward diversity, equity, and inclusion (DEI).
Step 2: Structured Mentorship Programmes	Develop formal mentorship networks connecting underrepresented nurses with senior leaders committed to inclusivity. Provide career coaching and sponsorship.	Increased career mobility, retention, and leadership readiness among diverse nurses.
Step 3: Leadership Development Pathways	Implement leadership training, professional development programmes, and policy advocacy initiatives. Ensure diverse candidate pipelines for nursing leadership roles.	Stronger representation of diverse nurse leaders across healthcare settings.
Step 4: Policy & Structural Reforms	Implement anti‐bias hiring practices, inclusive promotion criteria, and leadership accountability measures. Secure funding for DEI‐driven leadership initiatives.	Sustainable workforce transformation and equity‐centered institutional policies.
Step 5: Continuous Evaluation & Scaling	Conduct DEI audits, track leadership diversity metrics, and refine interventions based on data. Scale successful models to other institutions.	Long‐term systemic impact, ensuring equity‐driven leadership is embedded in practice.

### 4.6 Policy and Advocacy: Scaling the Framework for Systemic Change

The Equity‐Centered Nursing Leadership Framework (ECNLF) is not only a conceptual model for advancing diversity, equity, and inclusion in nursing leadership but also a call to action for systemic change. Policy advocacy plays a critical role in institutionalising these principles, ensuring that equity‐focused leadership development programmes become embedded within healthcare and academic institutions rather than remaining isolated initiatives. Scaling the framework requires aligning policy interventions with institutional reforms to overcome resistance, sustain leadership pipelines, and create long‐term workforce transformation (Iheduru‐Anderson and Alexander 2022).

One of the greatest barriers to sustaining diversity and inclusion initiatives is the lack of institutional policies that mandate workforce equity measures. Without policy enforcement, programmes such as CLCDN risk being underfunded, deprioritised, or discontinued due to leadership turnover or shifting institutional priorities (De Sousa and Varcoe 2022). Therefore, policy interventions are necessary to establish structural accountability that ensures equity‐driven leadership development remains integral to healthcare workforce planning.

At the federal and accreditation levels, legislative policies and professional standards have already demonstrated success in increasing workforce diversity. The Title VIII Nursing Workforce Diversity (NWD) Programme, for example, has provided financial support and leadership development opportunities for underrepresented nursing students, resulting in higher retention rates and career advancement outcomes (Health Resources & Services Administration [HRSA], 2024). Similarly, accreditation bodies such as The Joint Commission (TJC) and the American Nurses Credentialing Center (ANCC) now require cultural competency training, implicit bias education, and diversity‐focused hiring policies for healthcare institutions to maintain their credentials (TJC 2023; ANCC 2023). These standards underscore the importance of integrating workforce equity measures into institutional accreditation frameworks, reinforcing the need for policy advocacy at both the organisational and national levels.

At the institutional level, organisations that have successfully implemented diversity‐focused leadership development initiatives have done so by aligning them with policy reforms that mandate structured mentorship, anti‐bias training, and leadership coaching programmes (Bailey et al. 2017). For example, Mass General Brigham's Disparities Solutions Center has institutionalised anti‐bias hiring practices, leadership coaching for underrepresented nurses, and targeted mentorship pathways, leading to measurable increases in minority nurse retention and leadership representation (Hall et al. 2015). These efforts highlight how policy‐driven interventions can serve as a powerful mechanism for scaling the ECNLF across diverse healthcare and academic settings.

Furthermore, systemic policy change is necessary to address resistance to diversity, equity, and inclusion (DEI) initiatives within institutions. Resistance often stems from a lack of leadership commitment, resource constraints, and entrenched biases in hiring and promotion (Iheduru‐Anderson and Alexander 2022). To counteract this, advocacy efforts must focus on securing long‐term funding, integrating equity goals into institutional performance metrics, and holding leadership accountable for workforce diversity outcomes. Policy reforms that tie diversity metrics to funding eligibility, institutional accreditation, and executive compensation can ensure that leadership remains invested in workforce transformation efforts (Nikpour et al. 2022).

The scalability of the ECNLF ultimately depends on aligning nursing education, healthcare institutions, and policymakers in a coordinated effort to advance workforce equity. By leveraging legislative support, accreditation standards, and institutional policies, the ECNLF can transition from an innovative framework into an established standard for leadership development. Nursing schools and healthcare organisations must proactively engage in policy advocacy to ensure that equity‐centered leadership training and mentorship programmes become a permanent fixture in nursing workforce planning (Alasiri et al. 2024).

In summary, policy advocacy is the linchpin for sustainable systemic change in nursing leadership diversity. Without structured policy interventions, workforce equity efforts remain vulnerable to shifts in institutional priorities and funding limitations. By embedding DEI initiatives into federal policies, accreditation requirements, and institutional governance structures, the ECNLF can be scaled into a national model for nursing leadership transformation. Through intentional advocacy, strategic policy integration, and institutional commitment, nursing education and healthcare systems can ensure that workforce diversity is not merely an aspiration but a foundational principle of the profession.

### 4.7 Implications for Nursing

The Equity‐Centered Nursing Leadership Framework (ECNLF) and the Clinical Leadership Collaborative for Diversity in Nursing (CLCDN) offer valuable insights into transforming nursing leadership, education, and institutional policies. By integrating structured mentorship, equity training, and policy advocacy, the framework provides a replicable model for improving workforce diversity, leadership representation, and systemic change in nursing education and practice. The following implications highlight the necessity of embedding these principles within leadership development, workforce training, and policy frameworks to sustain long‐term diversity and equity improvements in the profession.

The ECNLF provides a structured approach to leadership development that prioritises mentorship, equity training, and policy advocacy as core strategies to increase leadership representation among underrepresented nurses. Leadership programmes that incorporate Black feminist and critical race perspectives ensure that nurses from marginalised backgrounds receive tailored support to navigate systemic barriers (Iheduru‐Anderson and Alexander 2022). By addressing disparities in leadership pipelines, these programmes help mitigate burnout, workplace dissatisfaction, and attrition rates among minority nurses (Bailey et al. 2017). Structured career advancement pathways such as leadership fellowships, executive training, and hospital‐based mentorship programs are essential for creating sustainable change. Programmes that offer targeted leadership coaching, networking opportunities, and sponsorship initiatives have been shown to accelerate the career progression of underrepresented nurses (Banister et al. 2020). Additionally, embedding diversity‐focused leadership development into healthcare organisational structures enhances retention, strengthens team dynamics, and improves patient care outcomes. By institutionalising equity‐centered leadership development, nursing organisations can build a sustainable model for increasing representation at the executive and managerial levels. Healthcare institutions, nursing schools, and professional associations must take a proactive role in integrating these principles into leadership development strategies to foster an inclusive and diverse nursing workforce.

Integrating equity‐centered leadership frameworks into nursing education is essential to preparing future nurse leaders to address workforce disparities and develop culturally responsive healthcare teams. Nurse educators must embed discussions on systemic racism, implicit bias, and intersectionality within leadership training programmes to cultivate culturally competent nursing professionals (De Sousa and Varcoe 2022). Educational institutions should expand career pipeline programmes that recruit, mentor, and support underrepresented nursing students throughout their academic and professional journeys. Programmes like the Nursing Workforce Diversity (NWD) Programme have demonstrated how financial aid, academic support, and leadership training can significantly improve workforce diversity and retention (Noone et al. 2020). Similar university‐healthcare partnerships, such as CLCDN, serve as successful models for increasing minority nurse participation in leadership roles (Guglielminotti et al. 2022). Additionally, nursing accreditation bodies should incorporate diversity, equity, and inclusion (DEI) training into educational standards, ensuring that all nursing programmes provide competency‐based learning experiences focused on equity‐driven leadership. Expanding faculty diversity and creating structured mentorship initiatives within nursing schools can also help reduce attrition among minority nursing students, ultimately fostering a more inclusive pipeline of future nurse leaders. By integrating ECNLF principles into nursing curricula, leadership training, and mentorship initiatives, nursing education programmes can proactively address disparities and equip nurses with the skills necessary to drive workforce equity.

Policy interventions play a crucial role in sustaining workforce diversity and leadership equity in nursing. Policymakers must: (1) expand funding for minority nurse leadership pathways, (2) mandate bias education and mentorship access in accreditation requirements, and (3) establish DEI accountability measures for healthcare organisations tied to federal funding. Institutional accreditation bodies such as the Joint Commission (TJC) and the Magnet Recognition Programme should also incorporate measurable diversity standards in leadership hiring and professional development programmes (TJC 2023). By establishing clear benchmarks for DEI initiatives, these accreditation agencies can hold healthcare institutions accountable for increasing minority representation in leadership and creating inclusive workplace environments. Healthcare organisations must implement data‐driven DEI audits to track disparities in hiring, promotion, and leadership development. Evidence‐based workforce assessments can identify gaps in representation, mentorship, and leadership opportunities, ensuring accountability in achieving diversity goals (Bailey et al. 2017). Additionally, organisations must integrate equity‐focused policies into executive leadership structures, requiring senior administrators to actively engage in diversity and inclusion efforts. To institutionalise sustainable change, policymakers, nursing schools, and healthcare leaders must collaborate to expand existing frameworks and integrate DEI policies into regulatory, funding, and leadership development structures. The ECNLF serves as a scalable model, demonstrating how mentorship, leadership training, and systemic policy interventions can drive workforce transformation and advance health equity across nursing practice.

## 5 Conclusion

The Equity‐Centered Nursing Leadership Framework (ECNLF) provides a structured, evidence‐based approach to addressing long‐standing inequities in nursing leadership and workforce diversity. By embedding mentorship, leadership development, and policy‐driven reforms, the framework ensures that workforce equity efforts are not temporary initiatives but sustained, institutionalised strategies. Diversity efforts in nursing must move beyond performative initiatives to systemic, sustained change that fosters an inclusive, representative, and equity‐driven workforce. To achieve this transformation, nursing schools must actively integrate equity‐centered leadership training into curricula, ensuring that future nurses are equipped with the skills, knowledge, and mentorship needed to navigate and dismantle systemic barriers. Healthcare institutions must take responsibility for implementing structured DEI initiatives, implicit bias training, and leadership pathways that create real opportunities for underrepresented nurses. Policymakers play a critical role in expanding funding for workforce diversity programmes and establishing accountability measures that require healthcare institutions to meet workforce equity benchmarks. Without deliberate and sustained efforts across education, healthcare, and policy sectors, racial and ethnic disparities in nursing leadership will persist, limiting career mobility, retention, and patient care outcomes.

The transformative impact of this framework is evident in the experience of LM, an underrepresented nursing student who, through participation in the CLCDN, received mentorship, leadership training, and institutional support that propelled them into a leadership role within a major healthcare system. LM's trajectory exemplifies how structured, equity‐centered initiatives create career mobility, empower diverse nurse leaders, and contribute to a more inclusive healthcare system. An equity‐centered nursing workforce cannot be built by a single institution or sector alone; it requires collective commitment and collaboration from educators, healthcare leaders, and policymakers. Addressing systemic barriers and fostering diversity in leadership must be recognised as a fundamental workforce priority, not an optional initiative. While ECNLF provides a roadmap, its long‐term effectiveness depends on its widespread adoption and continued evaluation. Future research should explore its impact across different healthcare systems to refine and sustain its implementation. The time for action is now. By scaling the ECNLF, expanding mentorship, strengthening policy‐driven leadership pathways, and ensuring institutional accountability, the nursing profession can cultivate a workforce that reflects and uplifts the diverse communities it serves.

## Author Contributions

The author made substantial contributions to conception and design, or acquisition of data, or analysis and interpretation of data; involved in drafting the manuscript or revising it critically for important intellectual content; given final approval of the version to be published. Each author should have participated sufficiently in the work to take public responsibility for appropriate portions of the content; agreed to be accountable for all aspects of the work in ensuring that questions related to the accuracy or integrity of any part of the work are appropriately investigated and resolved.

## Conflicts of Interest

The author declares no conflicts of interest.

## Data Availability

Data derived from public domain resources.
